# A Sequence in the loop domain of hepatitis C virus E2 protein identified *in silico* as crucial for the selective binding to human CD81

**DOI:** 10.1371/journal.pone.0177383

**Published:** 2017-05-08

**Authors:** Chun-Chun Chang, Hao-Jen Hsu, Jui-Hung Yen, Shih-Yen Lo, Je-Wen Liou

**Affiliations:** 1 Institute of Medical Sciences, Tzu Chi University, Hualien, Taiwan; 2 Department of Laboratory Medicine, Tzu Chi Medical Center, Hualien, Taiwan; 3 Department of Life Sciences, Tzu Chi University, Hualien, Taiwan; 4 Department of Molecular Biology and Human Genetics, Tzu Chi University, Hualien, Taiwan; 5 Department of Laboratory Medicine and Biotechnology, Tzu Chi University, Hualien, Taiwan; 6 Department of Biochemistry, School of Medicine, Tzu Chi University, Hualien, Taiwan; Academia Sinica, TAIWAN

## Abstract

Hepatitis C virus (HCV) is a species-specific pathogenic virus that infects only humans and chimpanzees. Previous studies have indicated that interactions between the HCV E2 protein and CD81 on host cells are required for HCV infection. To determine the crucial factors for species-specific interactions at the molecular level, this study employed *in silico* molecular docking involving molecular dynamic simulations of the binding of HCV E2 onto human and rat CD81s. *In vitro* experiments including surface plasmon resonance measurements and cellular binding assays were applied for simple validations of the *in silico* results. The *in silico* studies identified two binding regions on the HCV E2 loop domain, namely E2-site1 and E2-site2, as being crucial for the interactions with CD81s, with the E2-site2 as the determinant factor for human-specific binding. Free energy calculations indicated that the E2/CD81 binding process might follow a two-step model involving (i) the electrostatic interaction-driven initial binding of human-specific E2-site2, followed by (ii) changes in the E2 orientation to facilitate the hydrophobic and van der Waals interaction-driven binding of E2-site1. The sequence of the human-specific, stronger-binding E2-site2 could serve as a candidate template for the future development of HCV-inhibiting peptide drugs.

## Introduction

Hepatitis C virus (HCV) affects approximately 170 million people worldwide [1] and is one of the major causes of liver diseases, including chronic hepatitis, liver cirrhosis, and hepatocellular carcinoma [2]. The current standard treatments for HCV infection are combinations of pegylated IFN-α, ribavirin, RNA-dependent RNA polymerase, and NS3-NS4-NS5 protease inhibitors, which generally result in 67%–75% sustained viral response rates [3–6]. However, these treatments can induce various side effects, and the resistance of HCV to these treatments has been discovered [7–9]. As a result, there is a need for alternative strategies to treat HCV infections. A prophylactic vaccine may help to control the HCV pandemic, but developing a vaccine involves technical challenges because of the high sequence variability of the viral genomes [10]. An increased understanding of HCV has allowed further development of new entry inhibitors with direct-acting antiviral agents. However, the most critical problem in the development of novel approaches to treat or prevent HCV infections is the lack of detailed information on the interactions between the virus and its hosts at the molecular level. Current cell and animal model systems cannot elucidate the HCV infection in detail, because HCV is highly species-specific—it can only infect humans and chimpanzees—and is therefore difficult to culture *in vivo* and *in vitro*. Although studies using special cell based culture systems have provided important information toward the understanding of the HCV infection [11–14], these systems are still difficult to be applied to study the details of protein-protein interactions at molecular and sub-molecular levels. With bioinformatic approaches becoming powerful tools in biomedical studies, the application of these techniques should yield valuable information about the virus–host interactions, which could lead to the development of more effective treatments.

HCV is an enveloped virus containing a positive-sense single-stranded RNA. Its genome encodes a single polyprotein of approximately 3100 amino acids containing structural and nonstructural proteins. The structural protein includes the core protein, envelope glycoprotein 1 (E1), and envelope glycoprotein 2 (E2), while the nonstructural proteins comprise the p7 viroporins and the NS2, NS3, NS4A, NS4B, NS5A and NS5B protease [15,16]. The entry of HCV into a hepatocyte is a complicated process that requires coordinated interactions between the viral envelope proteins and host cell surface receptors, including CD81, scavenger receptor B1, claudin-1, occluding, EGFR, and membrane-bound cell kinases [17–19]. The two transmembrane glycoproteins E1 and E2 of HCV form a heterodimer, and are believed to be responsible for the recognition and binding to the host cell receptors, as well as for mediating the fusion of the virus envelope with host membranes, enabling the virus to enter cells [20,21].

HCV E2, consisting of the amino acids 384 to 746 of the polyprotein, is believed to play an important role in the initial stage of HCV binding to the host cells, and has been considered a crucial immunogenic vaccine target [22–24]. HCV E2 has also been identified as being a determinant factor for the species-specificity of HCV [25–27]. In 2013, the globular structure of the E2 core with amino acid residues 412 to 645 was resolved. This structure contains a central immunoglobulin-fold β sandwich flanked by two additional protein layers. The neutralizing antibody AR3C binds to a large part of the front layer, which consists of the loops, short helices, and β sheets [23].

The transmembrane human surface receptor CD81 has been identified as the binding target for HCV E2 [13,28–31]. CD81 comprises the cytoplasmic N- and C- terminals, a small extracellular loop, a large extracellular loop (LEL), four transmembrane domains (TM1–4) and a cytoplasmic loop. CD81 LEL, which is located between TM3 and TM4, plays a direct role in HCV infection through mediating E2 binding. The LEL domain contains five α–helices (A–E), in which the C to D helices with I181, I182, L185, and F186 may contribute to the viral recognition surface [32]. The blockage of the interaction between CD81 LEL and HCV E2 may also contribute to arresting liver cirrhosis progression [12]. A published study has suggested that the head hydrophobic residues of CD81, including V169, L170, I181, I182, L185, and F186, may be crucial for CD81 to bind to HCV E2 [33]. Other studies have suggested that the CD81 residues L162, K171, I181, I182, N184, F186 and D196 also interact with the HCV E2 protein [34–37]. These studies have indicated that the head domain of the CD81 LEL region may be the binding site of HCV E2. However, studies using neutralizing antibodies on E2/CD81 interactions have suggested that a variety of HCV E2 fragments, such as residues 412–423 [14], 434–446 [38], and 529–535 [39] might potentially interact with CD81. In addition, mutagenesis studies have also suggested that the HCV E2 residues, such as W420, Y527, W529, G530, D535, and G^436^WLAGLFY^443^ motif, were critical for the E2 binding to CD81 [40–43]. Although it is possible that many of the E2 residues are responsible for CD81 binding, the interactions between HCV E2 and the host CD81 at molecular levels, and the energetic aspects of the binding between the residues of HCVE2 and CD81, are still not fully understood.

Previous studies have indicated that the various HCV E2 discontinuous outer loops might be the binding sites for CD81 [22,23,38,44]. However, the detailed binding process remains unknown. Because E2 might be the determinant factor for species-specific binding [16,28,31,34], understanding and comparing the interactions between HCV E2 and CD81s from different mammalian species might elucidate the cause of this species-specificity and how the interactions affect the binding process. This information can aid the development of novel treatment approaches. Therefore, in this study, the structural differences between human and rat CD81s were compared *in silico*, and the interactions between HCV E2 protein and CD81s from humans and rats were investigated using molecular docking prediction. Subsequently, *in vitro* surface plasmon resonance (SPR) experiments and cellular assays were applied to validate the *in silico* results. This study developed a potential method of combining *in silico* molecular simulations and *in vitro* experiments to study complex virus–host interactions, to inform strategies for treating viral infections.

## Materials and methods

### Homology modeling

The structure of E2 in this study was mostly taken from the crystal structure of HCV E2 (PDB ID: 4MWF) [23]; however, there are breakpoints in the published crystal structure, and these structural breakpoints have been filled by homology modeling. Human CD81 has a crystal structure (PDB ID: 1IV5) [32,33]. Because there is currently no rat CD81 structure available in the PDB, the rat CD81 structure was constructed with homology modeling. The full-length structures for HCV E2 and rat CD81 were constructed using homology modeling supported in the Phyre2 web server (http://www.sbg.bio.ic.ac.uk/phyre2/) [45]. The homology modeling of the rat CD81 structure was performed by using the resolved human CD81 structure (PDB ID: 1IV5) as the template. The sequence identity between human and rat CD81s is over 93%, which makes the human CD81 an excellent template for modeling the rat protein. The breakpoint-free HCV E2 structure was constructed through homology modeling using the HCV E2 structure (PDB ID: 4MWF) [23] as the template. The homology modeling structures were refined by using molecular dynamics (MD) simulations for further analysis.

### Molecular docking

The initial favorable sites for HCV E2 binding to the human and rat CD81 receptors were determined using the “dock proteins protocol” (ZDOCK) from Discovery Studio 3.5 (Accelrys Inc., San Diego, CA). The ZDOCK protocol is used to conduct the rigid-body docking of two protein structures as well as clustering the poses according to the ligand position using a fast Fourier transformation and perform an exhaustive six-dimensional search in the translational and rotational space between the two molecules [46]. The ZRANK function, as part of the ZDOCK protocol, is used to re-rank the docked poses. The obtained complex configurations are ranked based on a scoring function of a linear-weighted sum of van der Waals (VDW) energies, electrostatics, and desolvation energies. Higher scores obtained from the ZDOCK program mean that the complex structures are of better quality. The RDOCK protocol can subsequently be used for further refinement of the dozens of poses with higher ZDOCK scores, using a CHARMm-based energy minimization scheme for the optimization of intermolecular interactions. Scoring is based on a CHARMm electrostatic energy term and a desolvation energy term [46]. The RDOCK scores are defined as the summation of the electrostatic energy from the predicted complex after minimization and the desolvation energy from the complex. The structure with the lowest RDOCK scores is selected for further MD simulations. In this study, we investigated the initial binding event of HCV E2 to human and rat CD81s using molecular docking. On the basis of previous studies regarding the interactions between CD81 and HCV E2 [13,17,31], two regions of HCV E2 (a.a. 421–446, and 519–535) were filtered to dock with human and rat CD81s. The molecular docking results were refined by using MD simulations to calculate the initial binding free energy for further analysis.

### Molecular dynamics simulations

All structures investigated in this study were solvated in a cubic water box by removing overlapping water molecules. After energy minimization, 100 mM of NaCl solution was added to neutralize the whole system. Each simulation ran for 50 ns. All MD simulations were carried out with GROMACS-4.5.5 software using a Gromos96 (ffG45a3) force field with an integration step size of 2 fs. The simulations were conducted with the *NPT* ensemble employing the velocity-rescaling thermostat at a constant temperature of 310 K, and 1 bar. The MD simulation protocol was followed. After energy minimization and equilibration, a 50 ns production run was carried out without any constraint on the complex structure.

### MM/PBSA binding free energy calculations

To determine the most stable complexes predicted by the molecular docking, the binding free energy (Δ*G*_*bind*_) for each complex was estimated by using the MM/PBSA approach exploited previously based on the snapshots extracted from the single trajectory of the complex (single trajectory method) [47–49]. The binding free energy of a ligand to a receptor in a solution is defined in the following equation:

$$\begin{array}{l} {\Delta G}_{\mathrm{bind}}=G_{\mathrm{complex}}-G_{\mathrm{receptor}}-G_{\mathrm{ligand}}\\ \;=\Delta H-T\Delta S+{\Delta G}_{\mathrm{solv}}\\ \;≅\Delta E_{\mathrm{MM}}-T\Delta S+{\Delta G}_{\mathrm{polar}}+{\Delta G}_{\mathrm{nonpolar}} \end{array}$$ (1)

where Δ*E*_*MM*_ is the change of the gas-phase MM energy, defined as

$${\Delta E}_{\mathrm{MM}}={\Delta E}_{\mathrm{internal}}+{\Delta E}_{\mathrm{elec}}+{\Delta E}_{\mathrm{vdw}}$$ (2)

where Δ*E*_*internal*_ indicates the bond, angle, and torsional angle energies, and Δ*E*_*elec*_ and Δ*E*_*vdw*_ denote the electrostatic and VDW energies, respectively. In Eq (1), Δ*G*_*solv*_ is the sum of electrostatic solvation energy (polar contribution), Δ*G*_*polar*_, and the nonelectrostatic solvation energy (nonpolar contribution), Δ*G*_*nonpolar*_. Δ*G*_*nonpolar*_ was considered proportional to the solvent accessible surface area (SASA):

$${\Delta G}_{\mathrm{nonpolar}}=\gamma ∙\mathrm{SASA}+\beta$$ (3)

−TΔ*S* is the change of the conformational entropy upon ligand binding, and was not calculated here due to the expensive computing cost and poor prediction accuracy. A total of 300 snapshots extracted from the last 30 ns of stable MD trajectory per system were used for calculating all energy terms.

### Synthetic peptides

Based on the *in silico* analysis, peptides were designed for experimental validations of the binding site predictions. Four peptides (p_E2-site1, p_E2-site2, p_H-CD81 and p_R-CD81) designed according to the ZDOCK program and MM/PBSA binding free energy calculations were chemically synthesized and purchased from GeneMark (GMbiolab Co., Ltd., Taiwan). The sequences of the peptides used in this study are listed in Table 1. The human and rat CD81 peptides, p_H-CD81 and p_R-CD81, and the HCV E2 peptides, p_E2-site1 and p_E2-site2, were derived from the results of molecular docking between ligand E2 and receptor CD81. Control and HCV E2 mutant peptides were also synthesized (S1 Table) for the SPR and cell based binding measurements to rule out the possible non-specific bindings in the investigations. The HCV E2 mutant peptides were designed based on the mutation studies [11,13].

**Table 1:** Summary of all synthesized CD81 and HCV E2 peptides.

Name	Peptide Sequence
**p_H-CD81**	^167^TSVLKNNLCPSGSNIISNLFKE^188^
**p_R-CD81**	^167^TAVLRNSLCPSSSNSFTQLLKE^188^
**p_E2-site1**	^422^INSTALNCNESLNTGWLAGLFYQ^444^
**p_E2-site2**	^521^RSGAPTYSWGANDTDVF^537^

### Surface plasmon resonance measurements

SPR experiments were conducted to measure the binding efficiency of the designed ligand and receptor peptides. The SPR measurements were performed using a Biacore T200 workstation (GE Healthcare, USA). Peptides p_H-CD81 and p_R-CD81 were diluted to a concentration of 7.5 μM in a 10 mM sodium acetate buffer at pH 4.0 and immobilized onto a CM-5 sensor chip using amine coupling (EDC-NHS) for 5 min at a flow speed of 5 μL/min. Approximately 1000 response units (RU) of p_H-CD81 and p_R-CD81 were immobilized on the chip. In this study, data on the kinetics and affinity of ligand-receptor binding were obtained by flowing several different concentrations of the p_E2-site1 and p_E2-site2 over the chip sequentially at a flow rate of 30 µL/min for 2 min. The response was measured at two time points, 0 and 120 sec, to obtain information on the rate of CD81 peptides association with and dissociation from the p-E2-site1 and p-E2-site2 peptides. Equilibrium binding curves were generated and fitted using a monovalent binding model for each peptide to determine the K_D_ of the binding of E2 peptides (p_E2-site1 and p_E2-site2) with receptor CD81 peptides (p_H-CD81 and p_R-CD81).

### Cell culture

The Huh-7 cells, obtained from the Bioresource Collection and Research Center (Hsinchu, Taiwan), were cultured in Dulbecco’s modified Eagle’s medium supplemented with 10% fetal bovine serum (FBS; Invitrogen, Carlsbad, CA, USA) at 37°C in a 5% CO_2_ incubator. The rat adrenal pheochromocytoma cell line, PC-12, was obtained from the Bioresource Collection and Research Center (Hsinchu, Taiwan) and maintained in a RPMI-1640 medium supplemented with 10% heat-inactivated horse serum (HS), 5% FBS (Invitrogen, Carlsbad, CA, USA), and 1% NEAA in a 5% CO_2_ incubator at 37°C.

### Assay of peptide/cell binding by flow cytometry

Cells (1 × 10^6^ cells/mL) in phosphate buffered saline (PBS) containing 5% bovine serum albumin (BSA) were treated with different concentrations (2, 5, 10, 20, 30, and 40 μM) of peptides (p_E2-site1 and p_E2-site2) labelled with the fluorescent dye 5-carboxyfluorescein, for 30 min in dark conditions. The cells were washed twice using PBS with 5% BSA before analysis by flow cytometry (BD FACSCalibur^TM^). Data were analyzed with the Windows Multiple Document Interface (WinMDI) program, version 2.8.

### Inhibition of anti-CD81 binding to the cells by E2 peptides

To prove the p_E2-site1 and p_E2-site2 peptides binding to Huh-7 cells are specific to the cell surface receptor CD81, we pretreated the cells (1 × 10^6^ cells/mL) with nonfluorescent p_E2-site1 and p_E2-site2 peptides at different concentrations (10, 20, and 40 μM) in PBS containing 5% BSA for 30 min. The pretreated cells were then treated with FITC-labelled anti–human-CD81 monoclonal antibodies for 30 min in the dark. Cells were washed twice with 5% BSA contained PBS before being measured using flow cytometry. Data were analyzed with WinMDI version 2.8.

## Results

### Construction and analysis of the rat CD81 structure

**Fig 1:**
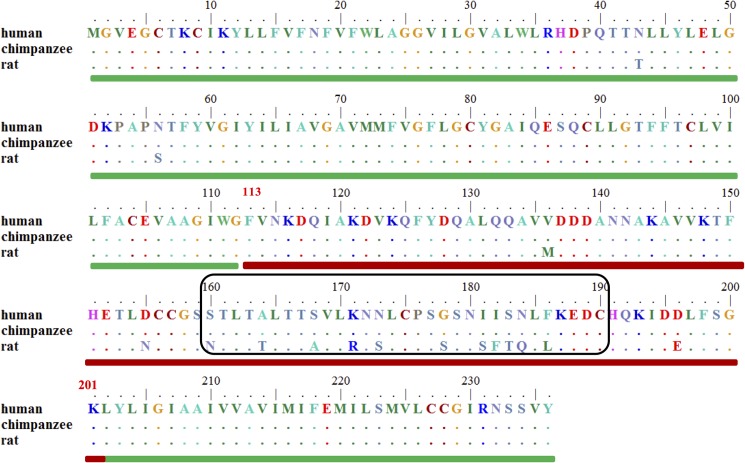
Sequence alignment of human, chimpanzee, and rat CD81s. The sequence alignments of full-length human and chimpanzee CD81s display 100% identity, whereas that of human and rat CD81s show over 93% identity (97% similarity). The sequence identity of the LEL between humans and rats is 84% (93% similarity) and that of the transmembrane domain is 99%, indicating that the major differences between human and rat CD81s are in the LEL. The transmembrane domain at a.a. 1–112 and 202–236 is indicated with a green rod, whereas the LEL region at a.a. 113–201 is indicated with a red rod. The largest differences between the CD81s located within 160–190 a.a are within the black–framed box.

**Fig 2:**
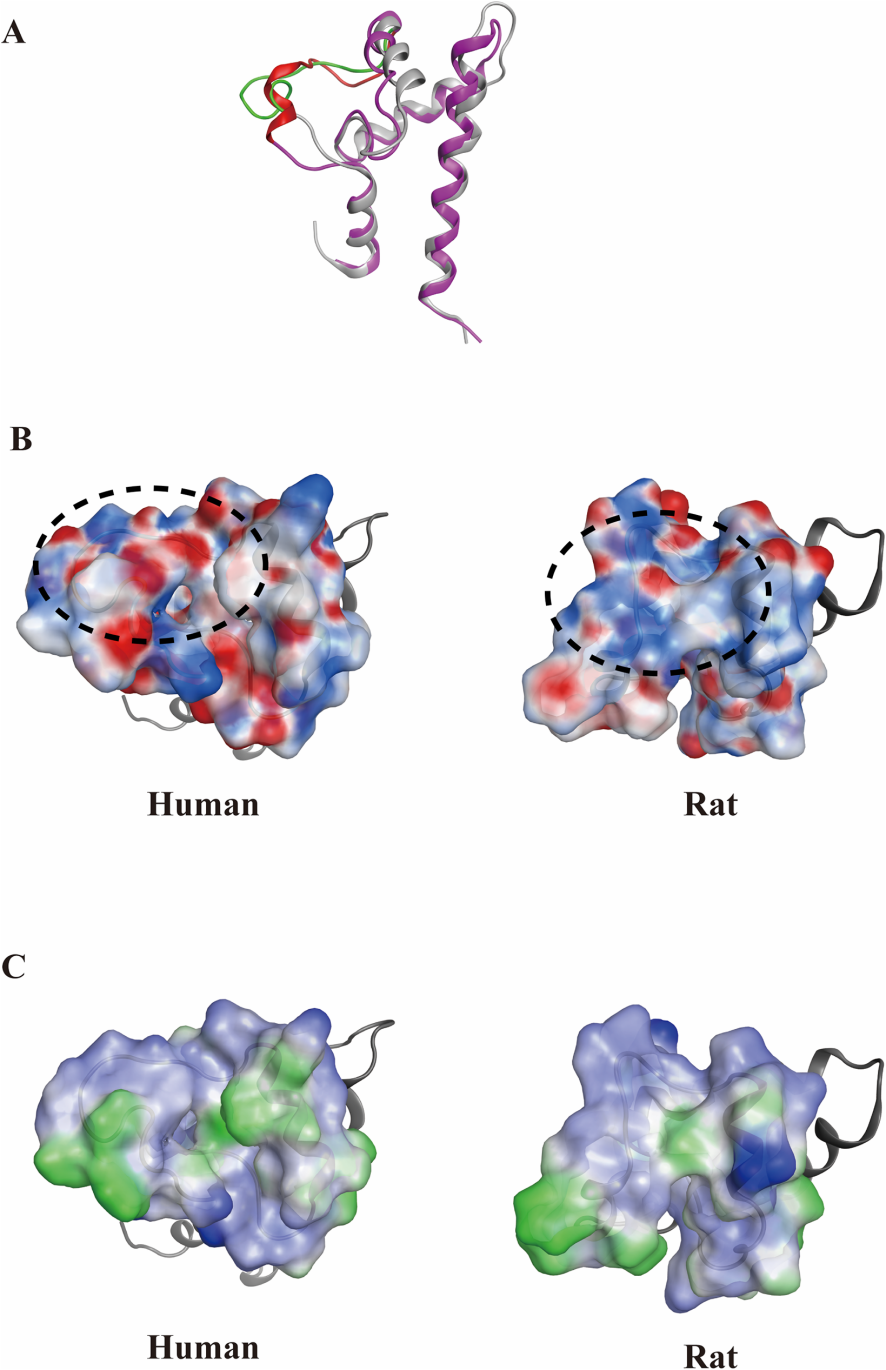
Comparison of surface charge and lipophilicity distributions between human and rat CD81 ectodomains. The structures of the resolved human CD81 (gray) and the homology-modeled rat CD81 (pink) shown in the ribbon were superposed. The major differences between the two CD81 structures are at the flexible loops from 173 to 186 a.a. (green: human CD81; red: rat CD81). (B) The surface charge distributions of the two CD81s show that the rat CD81 is more positively charged than human CD81 at the flexible loop region marked with a dashed line (blue: positive charge; red: negative charge). (C) The lipophilicity maps do not show much difference between the human and rat CD81s in the loop region (blue: hydrophilic; green: lipophilic).

The sequence alignments of full-length human and rat CD81s show over 93% identity, with the largest difference between them being located in a.a. 160–190 (Fig 1). Because of the high similarity between human and rat CD81s and the lack of rat CD81 structure, homology modeling was used to obtain the rat CD81 structure by applying the human CD81 structure (PDB ID: 1IV5) as a template. For homology modeling, if the sequence identity between the template and target proteins is more than 50%, the modeled structure should be reliable [50,51]. In our case, this was indicated by an extremely high sequence identity (over 93%) between human and rat CD81s. The modeled structure was also refined by running 50 ns MD simulations to obtain the equilibrated rat CD81 structure. The LELs of human and rat CD81 structures were superposed to analyze the electrostatics and hydrophobicity distributions by using the Molecular Operating Environment software (MOE 2014.09) (http://www.chemcomp.com). The major difference between the two structures is located at the flexible loop from residues 173 to 186, meaning that this region may be the cause of HCV E2 binding only to human CD81, but not to rat CD81 (Fig 2A). The loop region of the extracellular domain of human CD81 is flatter than that of rat CD81. The electrostatic distribution of the two CD81s shows that the rat CD81 is more positively charged than the human CD81 (Fig 2B). However, the hydrophobicity map of human CD81 does not exhibit much difference from rat CD81 in the loop region (Fig 2C).

### Molecular docking of human and rat CD81 to HCV E2

**Fig 3:**
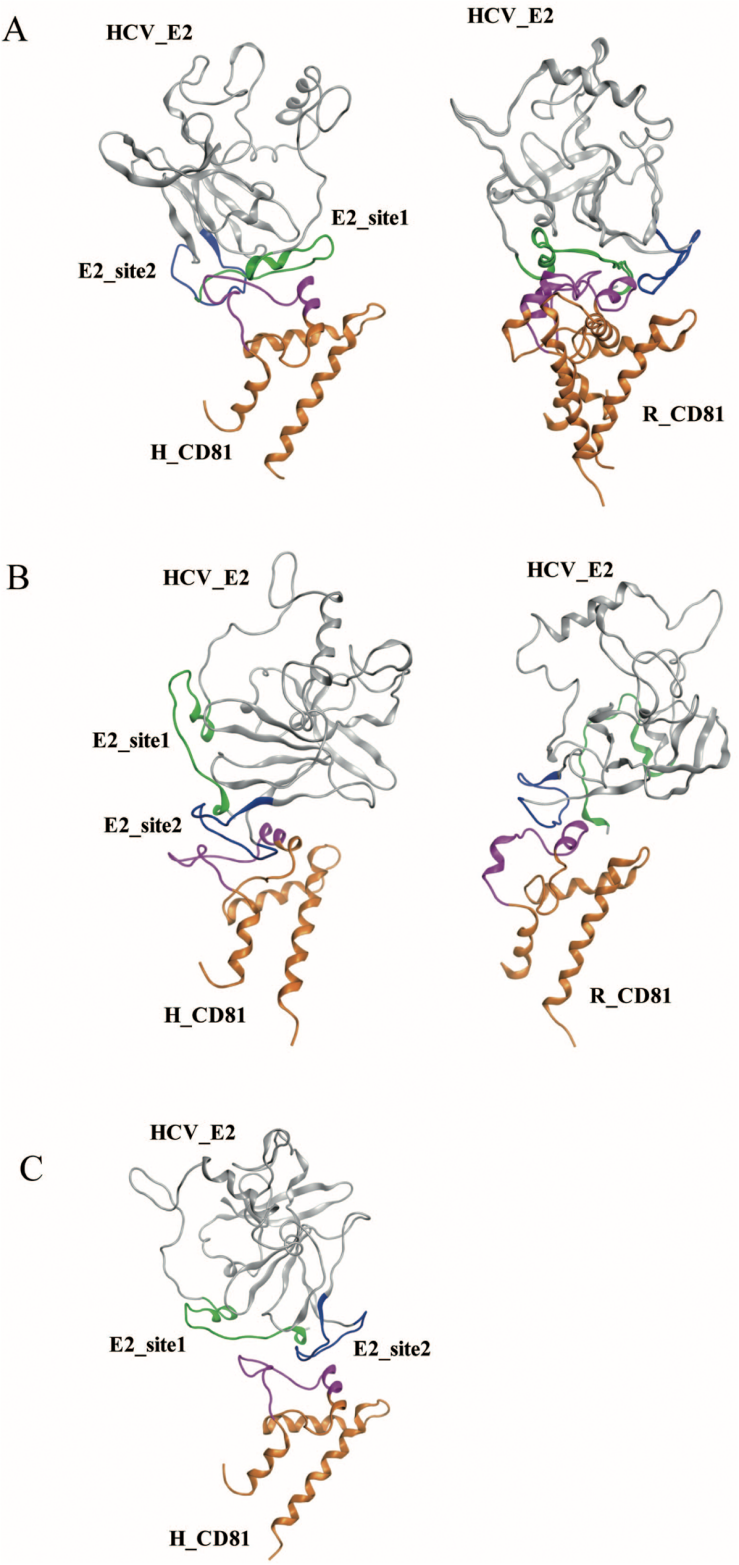
Molecular docking of human and rat CD81s to HCV E2 protein. Preferable sites of HCV E2 binding with human and rat CD81s are shown in A to C. (A) The HCV E2-site1 loop could bind to human and rat CD81s with similar RDOCK scores (human: −18.3 kcal/mol; rat: −16.2 kcal/mol). (B) The HCV E2-site2 loop was able to dock to human and rat CD81s, but the RDOCK score for human CD81 was more than twice as low as rat CD81 (human: −14.7 kcal/mol; rat: −6.2 kcal/mol). (C) HCV E2 bound to human CD81 with both E2-site1 and E2-site2 loops (RDOCK score: -19.1 kcal/mol). Green: E2-site1; blue: E2-site2; pink: the binding loops of human and rat CD81s.

The initial favorable sites for the HCV E2 binding to the receptor CD81s (human and rat) were determined based on the docking scores. The preferable docking results are shown in Fig 3. Although the HCV E2-site1 (a.a. 422–444) can bind to both the human and rat CD81s with similar RDOCK scores (human: −18.3 kcal/mol; rat: −16.2 kcal/mol), the binding orientations are quite dissimilar (Fig 3A). However, the RDOCK scores of the HCV E2-site2 (a.a. 521–537) binding with human CD81 is over twice as low than that with rat CD81 (human: −14.7 kcal/mol; rat: −6.2 kcal/mol), indicating that the HCV E2-site2 may not be favorable for binding to rat CD81 (Fig 3B). Finally, HCV E2 binding to human CD81 with both E2-site1 and E2-site2 exhibits a preferable binding pose (RDOCK score: -19.1 kcal/mol), whereas the docking results do not show any favorable pose for HCV E2 binding to rat CD81 with both HCV E2 sites (Fig 3C). According to these binding sites on the HCV E2-CD81 complex structures, the HCV E2-site1 region (a.a. 422–444) interacted with the head domain region of the human and rat CD81s (a.a. 167–188), whereas the HCV E2-site2 region (a.a. 521–537) only preferred to interact with the head domain region of human CD81 (a.a. 167–188). These results suggest that HCV E2-site2 might be a determining factor for HCV binding to human CD81.

### MM/PBSA binding free energy calculations of various HCV E2/CD81s binding complex structures

**Fig 4:**
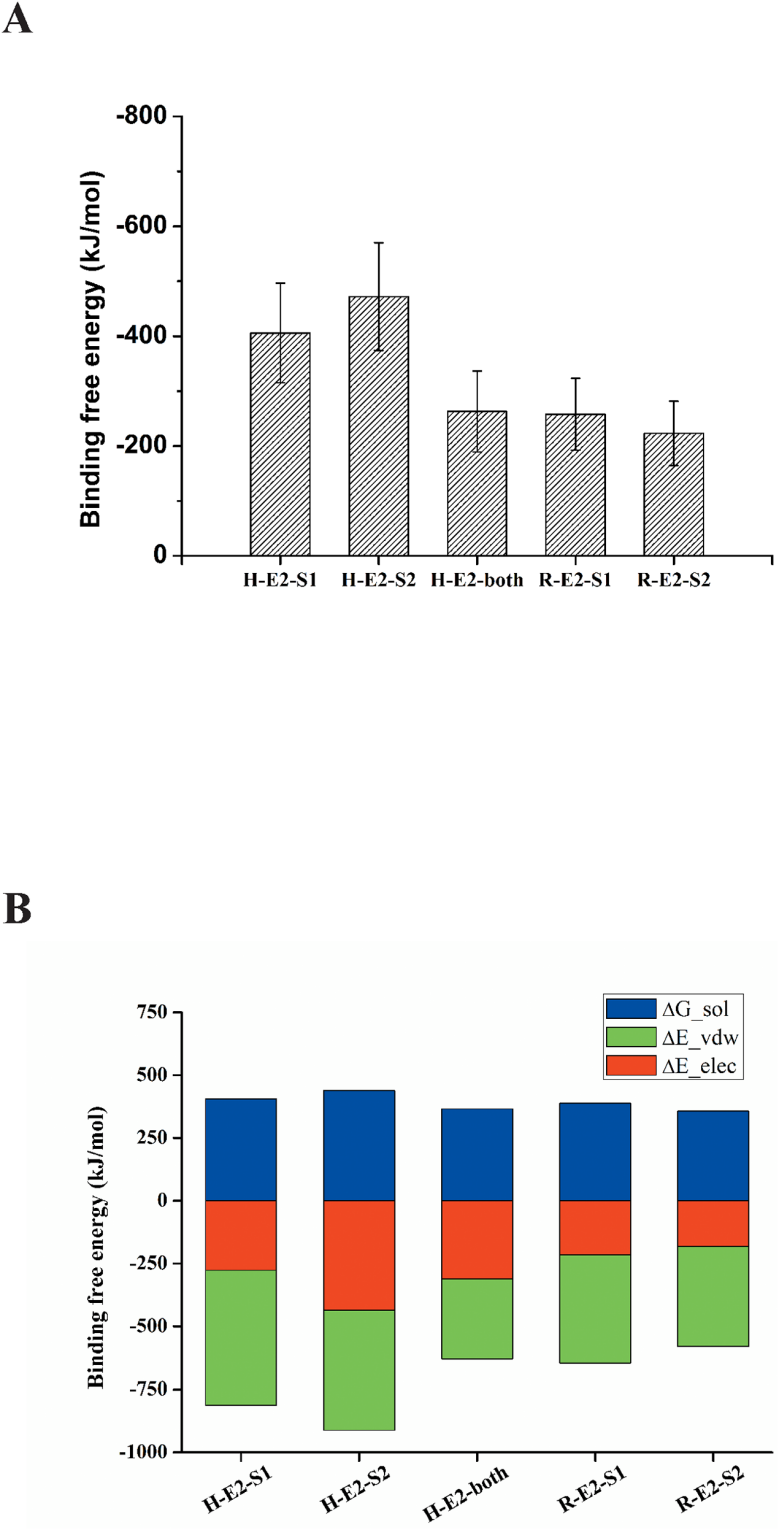
MM/PBSA binding free energy calculations for human and rat CD81s to HCV E2 protein. (A) For different HCV E2 sites (E2-site1, E2-site2, and E2-both sites) binding to human and rat CD81s, the binding free energies of human CD81 to HCV E2 were lower than those of rat CD81. HCV E2-site2 bound to human CD81 with the lowest binding free energy (H-E2-S2). (B) The detailed analysis of the components of binding free energies showed that the major difference for HCV E2-site2 binding to human and rat CD81s lies in the electrostatic interactions (H-E2-S2 and R-E2-S2). VDW dominates the binding of HCV E2-site1 to human CD81 (H-E2-S1). The figure represents the following. For H-E2-S1: the E2-site1 binding to human CD81; for H-E2-S2: the E2-site2 binding to human CD81; for H-E2-both: E2-both sites binding to human CD81; for R-E2-S1: the E2-site1 binding to rat CD81; and for R-E2-S2: the E2-site2 binding to rat CD81.

**Fig 5:**
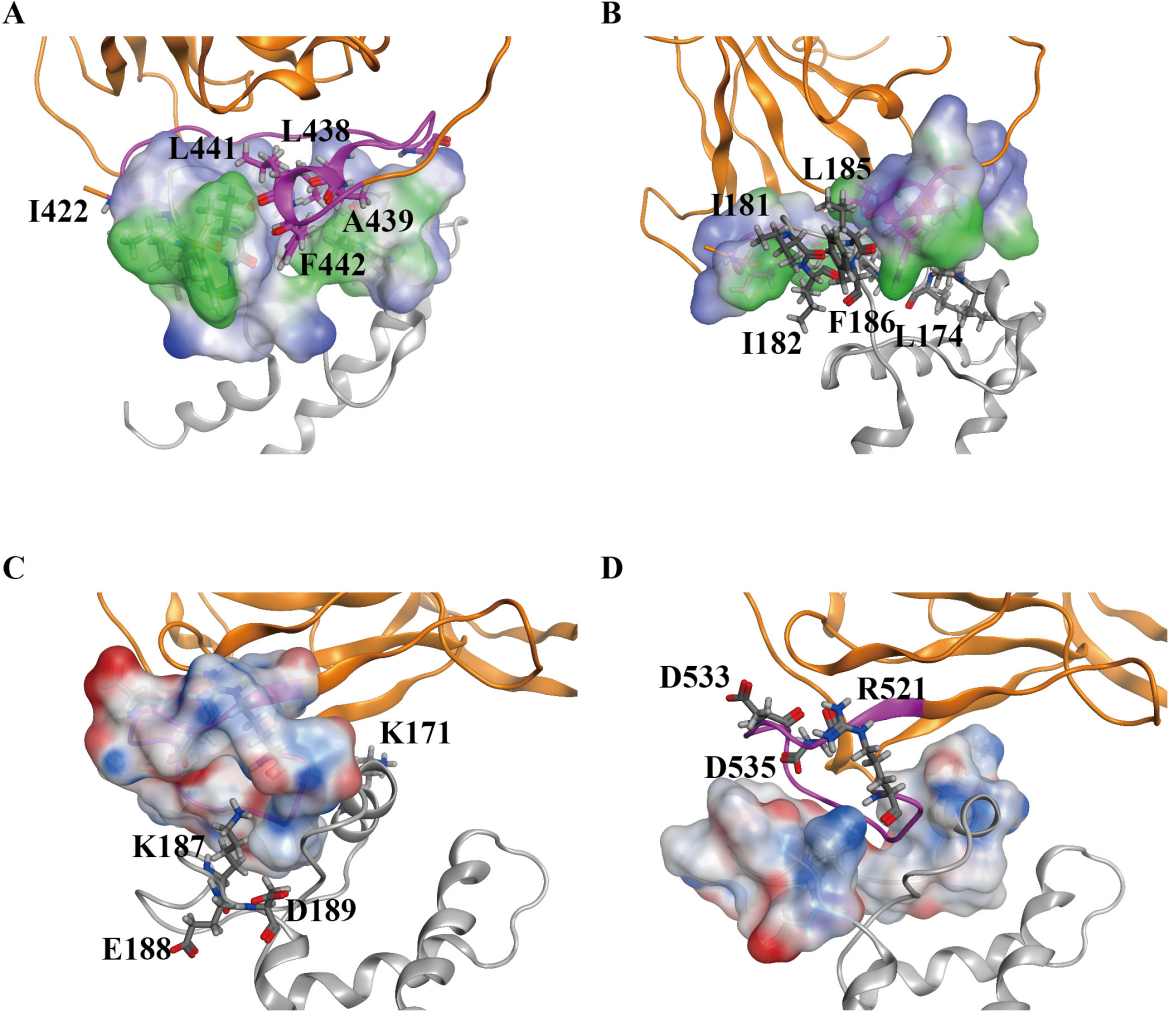
Surface charge and lipophilicity distributions for HCV E2 binding to human CD81. The complex structure is presented as a ribbon (orange: HCV E2; gray: human CD81). (A) and (B) are the surface lipophilicity distributions on HCV E2-site1 and human CD81 at the binding interface. In the figures, blue represents the hydrophilic part and green the hydrophobic part. The hydrophobic residues around the binding interface are labelled and presented as sticks. (C) and (D) are the surface charge distributions on HCV E2-site2 and human CD81 at the binding interface mapped according to the Poisson-Boltzmann equation. Blue and red correspond to positive and negative electrostatic potential, respectively. Charged residues around the binding interface are labelled and presented as sticks.

The most preferable poses for human and rat CD81s binding to HCV E2-site1 and E2-site2 were selected for further 50 ns MD simulations to calculate the initial binding free energies by the MM/PBSA program. Fig 4A shows that the binding free energies of HCV E2-site1 and E2-site2 to human CD81 (H-E2-S1 and H-E2-S2) were lower than those binding to rat CD81 (R-E2-S1 and R-E2-S2), and that the binding free energy of E2-site2 to human CD81 (H-E2-S2) (−472.1 kJ/mol) was over twice as low as that to rat CD81 (R-E2-S2; −223.2 kJ/mol), suggesting that the HCV E2-site2 loop favors selectively binding with human CD81. Notably, the respective binding free energies of HCV E2-site1 and E2-site2 to human CD81 (H-E2-S1 and H-E2-S2) were much lower than those of both E2 sites to human CD81 (H-E2-both), indicating that HCV E2 might not prefer to initially bind to human CD81 with both E2 sites simultaneously. Both the solvation energy and VDW interactions dominated the initial binding of HCV E2-site1 to human receptor CD81 (H-E2-S1), whereas in addition to solvation and VDW energies, electrostatic interactions also play an important role in HCV E2-site2 binding to human CD81 (H-E2-S2; Fig 4B). The major difference for HCV E2-site2 binding to human and rat CD81s (H-E2-S2 and R-E2-S2) was the contributions from the electrostatic interactions (Fig 4B), which may be the key determinant factor for the E2-site2 to bind to CD81. After 50 ns MD simulations, the final complex structures were taken for mapping the lipophilicity and surface charge distributions of the binding sites between HCV E2 and human CD81 (Fig 5). The lipophilicity map of E2-site1 binding to human CD81 showed that some hydrophobic residues of E2-site1, such as I422, L438, A439, L441, and F442, interact with the hydrophobic residues of the human CD81 binding loop (L174, I181, I182, L185, and F186). This is consistent with the MM/PBSA binding free energy calculations of H-E2-S1, in which VDW and solvation energies both dominate the initial binding (Figs 5A, 5B and 4B). Moreover, the surface charge distribution map of E2-site2 binding to human CD81 showed that charged residues between E2-site2 (R521, D533, and D535) and the human CD81 binding loop (K171, K187, E188, and D189) form a strong electrostatic field (Fig 5C and 5D). This result is also in accordance with the MM/PBSA binding free energy calculations of H-E2-S2, in which electrostatic interactions play a crucial role in the binding (Figs 5C, 5D and 4B).

### SPR measurements of the interactions between the derived peptides from HCV E2 and human and rat CD81s

**Fig 6:**
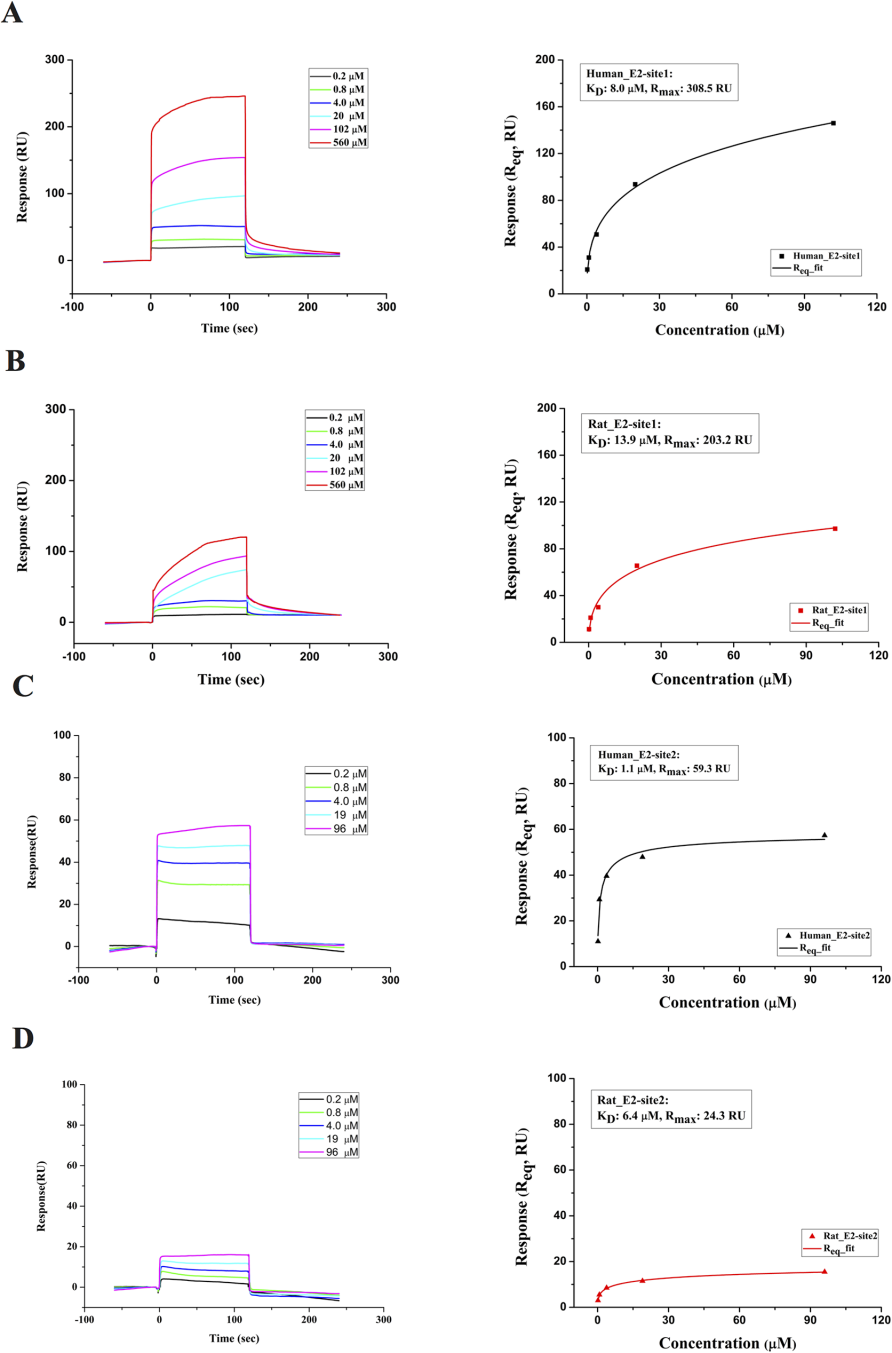
SPR measurements for the interactions between peptides derived from HCV E2 and CD81s. (A) SPR responses when HCV p_E2-site1 peptide in various concentrations was flowed over the immobilized human CD81 peptide (left). The equilibrium K_D_ of 7.96 ± 1.7 μM for p_E2-site1 binding to human CD81 peptide was determined by steady-state interaction isotherm (right). (B) SPR responses measured when p_E2-site1 in various concentrations was tested on rat CD81 peptide (left). The equilibrium K_D_ of 13.85 ± 2.46 μM for the p_E2-site1 binding to rat CD81 peptide was given by the binding isotherm (right). (C) SPR response when peptide p_E2-site2 in different concentrations was flowed over the immobilized human CD81 peptide (left). The binding isotherm gives the equilibrium K_D_ of 1.07 ± 0.09 μM for p_E2-site2 binding to human CD81 peptide (right). (D) SPR responses when peptide p_E2-site2 in various concentrations was tested on rat CD81 peptide. The response only increased slightly as the concentration of the peptide increased (left). The equilibrium K_D_ of 6.38 ± 0.58 μM for the p_E2-site2 binding to rat CD81 peptide was calculated from the steady-state binding isotherm (right). The K_D_ values shown are the averages of three measurements. Errors for K_D_ are standard deviations.

Based on the *in silico* analysis, the four peptides p_H-CD81, p_R-CD81, p_E2-site1 and p_E2-site2 (sequences shown in Table 1) were chemically synthesized for SPR measurements. The SPR results of binding affinity between the ligand peptides from HCV (p_E2-site1 and p_E2-site2) and the receptor peptides from the head domain region of human and rat CD81s (p_H-CD81 and p_R-CD81) are shown in Fig 6. For steady-state interactions, binding isotherms were created to determine the equilibrium dissociation constants (K_D_) for each interaction. According to SPR sensorgrams, it seems that the ligand peptide p_E2-site1 was able to bind to both of the receptor peptides p_H-CD81 and p_R-CD81 in a dose-dependent manner (Fig 6A and 6B). However, the p_E2-site1 binding to the rat peptide had a considerably higher K_D_ (13.85 ± 2.46 μM) than that of binding to the human peptide (7.96 ± 1.7 μM), indicating that the p_E2-site1 was less favorable for binding to the rat peptide than the human one. However, the p_E2-site2 peptide was found to bind only to p_H-CD81 (Fig 6C and 6D). The kinetic analysis of the binding isotherm showed that the K_D_ for p_E2-site2 to p_R-CD81 (6.38 ± 0.58 μM) was approximately sixfold higher than that of the peptide binding to p_H-CD81 (1.07 ± 0.09 μM; Fig 6C and 6D). The SPR results show that both of the E2 peptides bind better to the human CD81 peptide than to the rat one. The binding of p_E2-site2 to human CD81 peptide also has the lowest K_D_ indicating that the p_E2-site2 might be the determining factor for the binding of HCV E2 to human CD81. The possibility of non-specific bindings in the SPR experiments was ruled out by the no response results in the tests using control and E2 mutant peptides (S1 Fig).

### HCV E2 peptides binding to CD81 expressing human and rat cells

**Fig 7:**
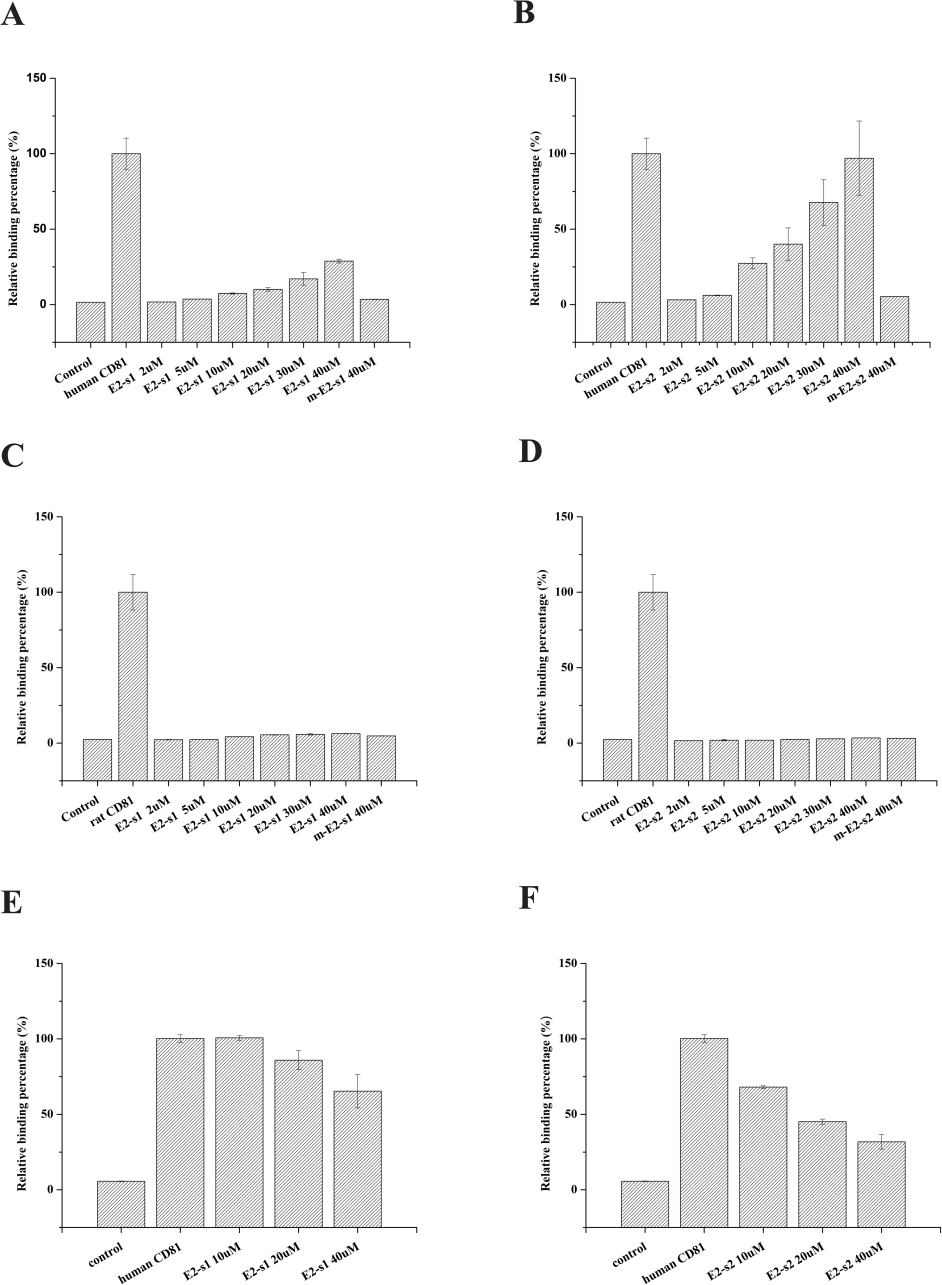
Flow cytometry of E2 peptides binding to human and rat cells and inhibitions of anti-CD81 antibody/cell binding by E2 peptides. (A) and (B) show the fluorescence intensity of Huh-7 cells treated with fluorescent p_E2-site1 (E2-s1) and p_E2-site2 (E2-s2), respectively, at different concentrations; (C) and (D) are the fluorescence intensity measurements of the rat PC12 cells treated with fluorescent p_E2-site1 or p_E2-site2, respectively; and (E) and (F) are the inhibitions of fluorescent anti-CD81 antibodies targeting Huh 7 cells by HCV E2 peptides. In this experiment, untreated cells were used as negative controls, and the cells treated with fluorescent-labelled anti-CD81 antibodies were used as positive controls and set as 100% binding. p_m_E2-site1 (m-E2-s1) and p_m_E2-site2 (m-E2-s2) in (A), (B), (C) and (D) are mutant peptides of p_E2-site1 and p_E2-site2 indicated in S1 Table.

Following the peptide/peptide binding measurements using SPR, peptide/CD81 protein binding assays on cells were carried out using flow cytometry (representative fluorescence histograms are shown in graphs A to D in S2 Fig, and quantitative statistical data are shown in Fig 7A–7D). In this study, Huh-7 cells and PC-12 cells were used to represent human and rat cells. The presence of CD81 on the cells was checked by fluorescent anti–human-CD81-FITC and anti–rat-CD81-FITC antibodies.

The results show that the increases in fluorescence intensity in both p_E2-site1- and p_E2-site2-treated Huh-7 cells occur in a dose-dependent manner, indicating that the peptides are able to bind to the cells. Furthermore, the p_E2-site2-treated Huh-7 cells had a higher fluorescence intensity than those treated with p_E2-site1 at the same concentrations (Fig 7A and 7B), indicating that the p_E2-site2 binds to Huh-7 cells better than p_E2-site1. However, the PC-12 cells showed no significant increase in fluorescence when treated with both p_E2-site1 and p_E2-site2, even at high concentrations (Fig 7C and 7D), indicating that the two peptides were not able to significantly bind to the rat PC-12 cells. To rule out possible non-specific binding in the experiments, tests on fluorescent-labeled E2 mutant peptides listed in S1 Table were performed. According to the tests, no significant increase in cell fluorescence was observed as fluorescent E2 mutant peptide concentration increased in all groups (S3 Fig). The quantitative statistical data of the mutant peptides binding at 40 μM (the highest concentration tested) are also shown in Fig 7A to 7D. The no binding results of the mutant peptides indicated that the positive binding results in the cell experiments were indeed specific.

### HCV E2 peptides inhibit CD81 antibodies binding to Huh 7 cells

To confirm that the p_E2-site1 and p_E2-site2 peptides binding to Huh-7 cells were indeed binding to CD81, we pretreated the cells with nonfluorescent p_E2-site1 and p_E2-site2 peptides, and then the fluorescent anti-CD81 antibodies were used to target CD81 on the cells. If the HCV E2 peptides specifically target CD81 on the cell surfaces, the pretreatments of the cell with the peptides should inhibit the binding of the fluorescent anti-CD81 antibodies to the cells, and thus the fluorescence of the antibody-treated cells should be reduced. The fluorescence of the cells was measured by flow cytometry (representative fluorescence histograms are shown in graphs E and F of S2 Fig, and quantitative statistical data are shown in Fig 7E and 7F). Both p_E2-site1 and p_E2-site2 were able to inhibit anti–human-CD81-FITC antibody binding to Huh-7 CD81, indicating that the bindings of the HCV E2 peptides were indeed specific to CD81 (Fig 7E and 7F). However, the p_E2-site2 peptide inhibited the antibody binding to Huh-7 cells at lower concentrations, meaning that the binding of p_E2-site2 to the cells was greater than that of p_E2-site1. This result agreed with our SPR measurements, in which the p_E2-site2/p_H-CD81 complex had a lower K_D_ value compared to that of p_E2-site1/p_H-CD81.

## Discussion

HCV binds to human cells with high specificity through the interactions between its E2 protein and host cell receptor CD81 [17,52], and humans and chimpanzees are the only known species that can be infected by HCV [32,33]. Previous studies have shown that humanized mice expressing only two human proteins, CD81 and occluding protein, can harbor the full life cycle of HCV [53,54], indicating that CD81 is a dominant factor for the species-specific recognition of HCV. However, the intracellular and transmembrane domains of CD81 are highly conserved among separate species, and the distinctions between CD81s from various species are very small. The CD81 sequence identity between human and rat is 93%, and 100% between human and chimpanzee. The tiny sequence variations in CD81s of separate species may be the key factor that decides whether HCV E2 is able to bind to their cells. Sequence analysis shows that the major differences between human and rat CD81 are located in the LEL region; otherwise, the transmembrane domains of human and rat CD81 are almost identical (Fig 1). We performed sequence alignment, surface charge distribution and lipophilicity map comparisons between human and rat CD81s and found the largest difference in the head domain of the CD81 LEL region (a.a. 167−188). This region was also identified by the molecular docking in our study as the binding region for HCV E2. This identified binding region is consistent with that proposed by other studies with various identification methods [25,32,33,36]. The molecular docking performed in this study employed the structures of HCV E2 and human and rat CD81s from X-ray crystallography and homology modeling. Based on the simulation results of molecular docking and binding free energy calculations for the interactions between HCV E2 and human and rat CD81s, two sequences ^422^INSTALNCNESLNTGWLAGLFYQ^444^ (p_E2-site1) and ^521^RSGAPTYSWGANDTDVF^537^ (p_E2-site2) on the HCV E2 protein were identified as responsible for CD81 binding. The results of our *in silico* identifications of HCV E2/CD81 binding sites agree with those proposed by several previous experimental studies. Neutralizing antibodies suggested that residues 434–446 [38] and 529–535 [39] potentially interact with CD81, which is very close to our finding. Mutagenesis studies on CD81/E2 interactions found that the E2 residues A524, P525, Y527, W529 [13] and E431, G523, G530, D535 [11] are important for CD81 binding. These residues are located well within the regions identified by our *in silico* calculations. The *in silico* identified sequences were then chemically synthesized for the SPR measurements for simple validations, and the SPR results were in accordance with our simulation results. The *in silico* and SPR comparison results showed that both sets of residues 422–444 and 521–537 bind better to human CD81 and derived peptide than to their rat counterparts. Furthermore, our results indicate that the residues 521–537 bind much better than the residues 422–444 to the human CD81, as indicated by the lower free energy from the calculations and validated by the lower K_D_ in the SPR measurements. Considering the huge differences in binding free energy and K_D_ values of the residues 521–537 binding to human and rat CD81 proteins/peptides, we suggest that the residues 521–537 (E2-site2) are the determinant factor for the HCV E2 to species-specifically bind to human CD81. As a result, in addition to proposing E2 fragments for CD81 binding, our study provides information concerning the binding affinity of E2 fragments to CD81s and the interactions involved in the HCV E2/CD81 binding at molecular levels.

This study also demonstrates the binding results of the two peptides derived from HCV E2 onto whole CD81 proteins on human and rat cells. The experiments revealed that neither of the HCV E2 peptides could bind to the rat cells expressing CD81. Although the SPR experiments on peptide/peptide interactions showed a possible weak binding (higher K_D_) of the E2-site1 on CD81 peptide, the interactions of the E2-site1 on whole rat CD81 were not observed. This indicates that the weak binding measured in simple peptide/peptide interaction systems might be prevented by other factors or parts of the whole proteins in more complicated systems such as peptide/protein-on-cells binding. However, both of the peptides derived from HCV E2 were able to significantly bind to the CD81 expressing human cells. The inhibition effects of the peptides on anti-CD81 antibodies targeting the CD81-expressing cells indicate that the binding of HCV E2 peptides on the cells might interrupt the epitope binding of the CD81 monoclonal antibody. Furthermore, the cell experiments clearly showed that the E2-site2 peptide exhibited greater binding efficiency and a better inhibition effect than the E2-site1 peptide at the same concentrations, again indicating that the E2-site2 might be dominant in HCV E2/CD81 binding.

**Fig 8:**
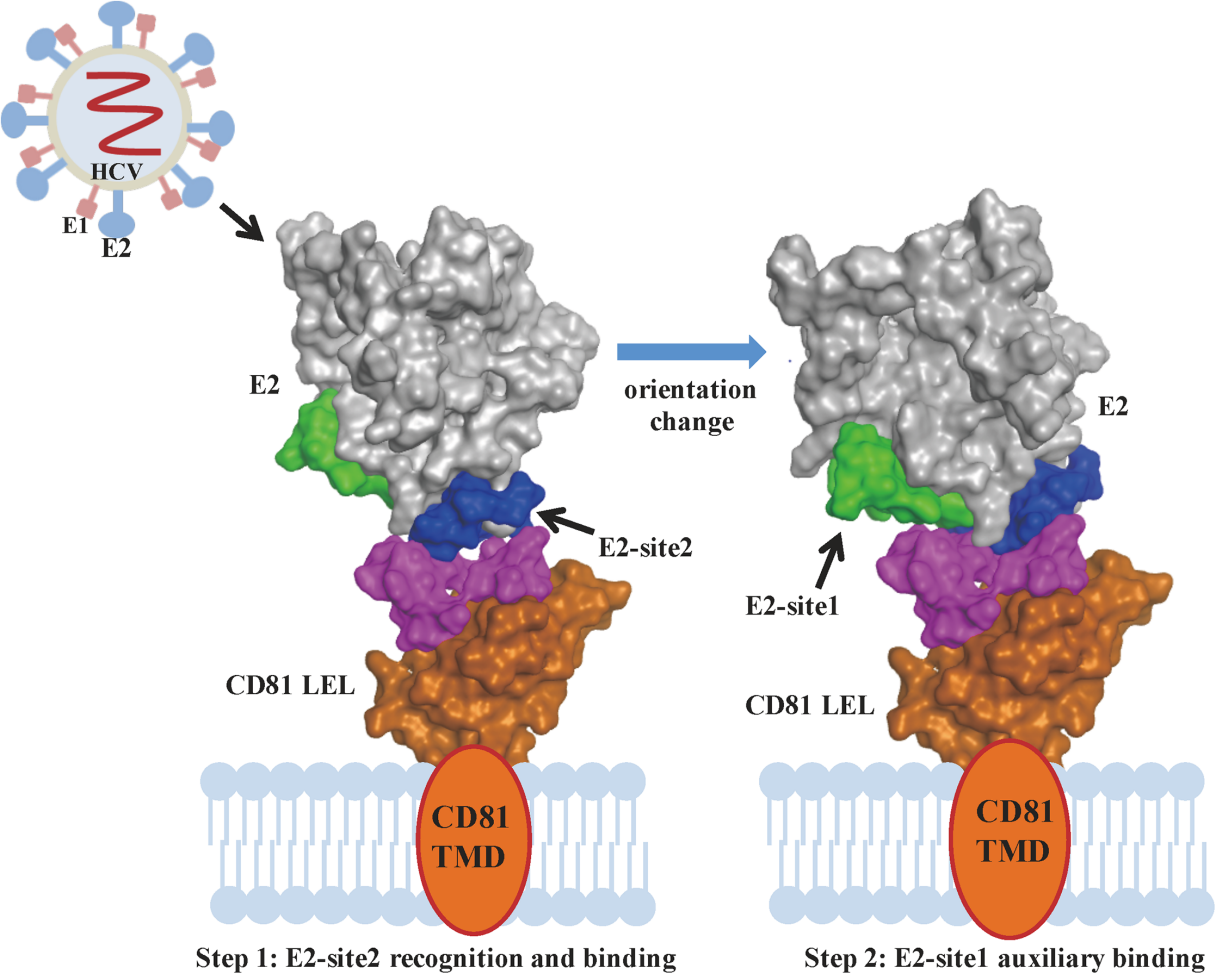
Putative model of the HCV E2/CD81 binding process. The initial HCV E2/CD81 binding process can be divided into two steps. Step 1: E2 initially recognizes and approaches CD81 with the E2-site2 region. Step 2: The orientation of E2 changes to a more preferable binding pose with the E2-site1 region auxiliary binding to execute the processes that follow. The E2-site1 region, E2-site2 region, and CD81 binding loop are presented in green, blue, and pink, respectively.

In this study, we proved that the tiny differences between the human and rat CD81s play a significant role in the infection specificity of HCV. *In silico* calculations were applied to study the interactions of HCV E2 protein to human and rat CD81s, and two regions that are important for the interactions—namely the E2-site1 and E2-site2—were identified. All the calculations and experiments demonstrated that these two regions interact powerfully with human CD81 than its rat equivalent, and that E2-site2 interacts with human protein more effectively than E2-site1. According to our *in silico* calculations, for HCV E2 to approach and bind to its target receptor with both binding sites simultaneously is unfavorable (the binding free energy for this condition is not the lowest). The binding of E2-site2 to CD81 has the lowest binding free energy and the highest affinity measured in the binding assay; therefore, the E2-site2 should be the first to bind. As a result, in addition to providing molecular information concerning the CD81/E2 binding sites, we provided a putative binding model of CD81/HCV E2. The binding might happen in two steps (Fig 8). In the first step, E2 recognizes and approaches the human CD81 with the host species-determinant E2-site2 residues from the dominant electrostatic interactions; in the second step, the orientation changes to a more preferable binding pose with the E2-site1 region auxiliary binding, driven by hydrophobic and VDW interactions. Many therapeutic agents are currently being developed to block HCV entry to hepatocytes, such as neutralization antibodies, peptide drugs, and small compounds [3,5,12]. Because of their relative low cost, minimal side effects, low viral resistance, and ease of use in combination therapy, small peptide drugs can be suitable and novel therapeutic candidates for inhibiting HCV E2 from binding to CD81. As demonstrated in this study, the binding of the human-specific E2-site2 onto CD81 is strong, and the binding of the E2-site2 peptide was even able to block the binding of the anti-CD81 antibody to the cells. The peptide derived from E2-site2 should serve as a suitable template to be developed into effective peptide drugs for interrupting HCV E2/host cell interactions. This study demonstrates a method for combining molecular simulations and simple *in vitro* experiments to investigate the initial events of a virus binding specifically to its host. The increased understanding of HCV–host interactions at the molecular level should benefit further developments of antiviral agents.

## Supporting information

S1 Fig SPR measurements for the interactions between control (random) or HCV E2 mutant peptides and CD81 peptides. (A) SPR responses measured when p_random-25 peptide in various concentrations was flowed over the immobilized human CD81 peptide. (B) SPR responses measured when p_m_E2-site1 in various concentrations was flowed over the immobilized human CD81 peptide. (C) SPR response measured when peptide p_random-18 in different concentrations was flowed over the immobilized human CD81 peptide. (D) SPR responses measured when peptide p_m_E2-site2 in various concentrations was flowed over the immobilized human CD81 peptide. (E) SPR responses measured when p_random-25 peptide in various concentrations was flowed over the immobilized rat CD81 peptide. (F) SPR responses measured when p_m_E2-site1 in various concentrations was flowed over the immobilized rat CD81 peptide. (G) SPR response measured when peptide p_random-18 in different concentrations was flowed over the immobilized rat CD81 peptide. (H) SPR responses measured when peptide p_m_E2-site2 in various concentrations was flowed over the immobilized rat CD81 peptide. As can be seen from these measurements, the SPR responses were not significantly increased with the increased concentrations of the control and mutant peptides.
(TIFF)

S2 Fig Representative fluorescence histograms of flow cytometry of HCV E2 peptides binding on human and rat cells, and inhibitions of anti-CD81 antibody/cell binding by E2 peptides. For various peptide concentrations, (A) shows the binding of fluorescent p_E2-site1 on Huh-7 cells, (B) shows the binding of fluorescent p_E2-site2 on Huh-7 cells, (C) shows the binding of fluorescent p_E2-site1 on rat PC12 cells, (D) shows the binding of fluorescent p_E2-site2 on rat PC12 cells, (E) shows the fluorescence histograms of the inhibitions of fluorescent anti-CD81 antibodies targeting Huh 7 cells by p_E2-site1 peptides, and (F) shows the fluorescence histograms of the inhibitions of fluorescent anti-CD81 antibodies targeting Huh 7 cells by p_E2-site2 peptides. In all experiments, untreated cells were used as negative controls (−C), and the cells treated with fluorescent-labelled anti-CD81 antibodies were used as positive controls (+C).
(TIFF)

S3 Fig Representative fluorescence histograms of flow cytometry of mutant HCV E2 peptides Binding on Human and Rat Cells. For various peptide concentrations, (A) shows the binding of fluorescent p_m_E2-site1 on Huh-7 cells, (B) shows the binding of fluorescent p_m_E2-site2 on Huh-7 cells, (C) shows the binding of fluorescent p_m_E2-site1 on rat PC12 cells, (D) shows the binding of fluorescent p_m_E2-site2 on rat PC12 cells. In all experiments, untreated cells were used as negative controls (−C), and the cells treated with fluorescent-labelled anti-CD81 antibodies were used as positive controls (+C). No dose dependent increase in fluorescence on the cells was observed when the fluorescent mutant peptides were added in different concentrations, indicating the mutant peptides were not able to bind to the CD81 presenting cells.
(TIFF)

S1 Table Summary of synthesized control (random) and HCV E2 mutant peptides. The mutated amino acids in the HCV E2 mutant peptides (p_m_E2-site1 and p_m_E2-site2) are designed based on the mutation studies [11,13].
(DOCX)
