# Supplementary figures and images for: A Sequence in the loop domain of hepatitis C virus E2 protein identified in silico as crucial for the selective binding to human CD81
Source: PLoS One. 2017 May 8;12(5):e0177383. doi: 10.1371/journal.pone.0177383 (PMC5421814; doi:10.1371/journal.pone.0177383)

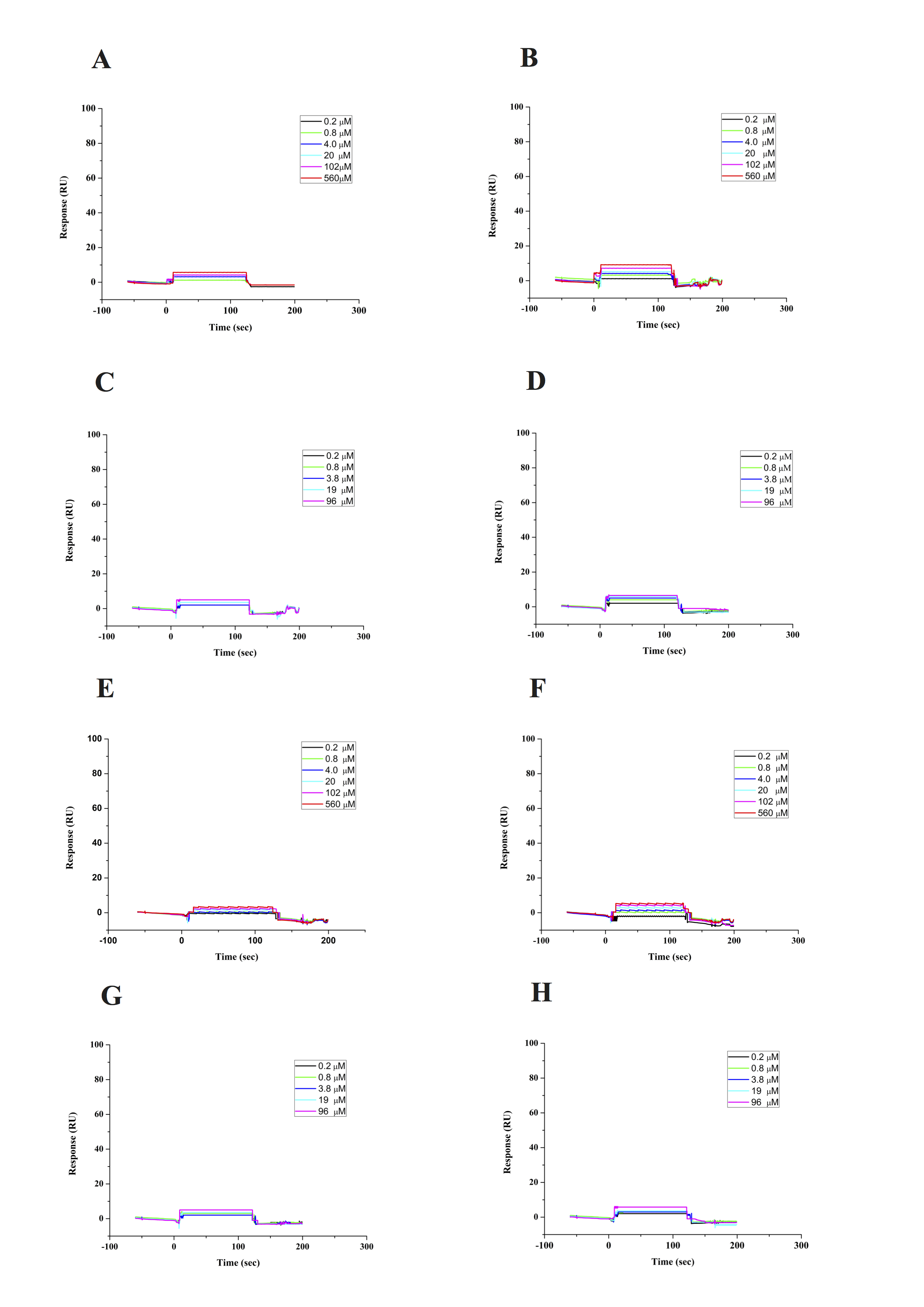

Supplement: S1 Fig — (A) SPR responses measured when p_random-25 peptide in various concentrations was flowed over the immobilized human CD81 peptide. (B) SPR responses measured when p_m_E2-site1 in various concentrations was flowed over the immobilized human CD81 peptide. (C) SPR response measured when peptide p_random-18 in different concentrations was flowed over the immobilized human CD81 peptide. (D) SPR responses measured when peptide p_m_E2-site2 in various concentrations was flowed over the immobilized human CD81 peptide. (E) SPR responses measured when p_random-25 peptide in various concentrations was flowed over the immobilized rat CD81 peptide. (F) SPR responses measured when p_m_E2-site1 in various concentrations was flowed over the immobilized rat CD81 peptide. (G) SPR response measured when peptide p_random-18 in different concentrations was flowed over the immobilized rat CD81 peptide. (H) SPR responses measured when peptide p_m_E2-site2 in various concentrations was flowed over the immobilized rat CD81 peptide. As can be seen from these measurements, the SPR responses were not significantly increased with the increased concentrations of the control and mutant peptides. (TIFF) [file pone.0177383.s001.tiff]

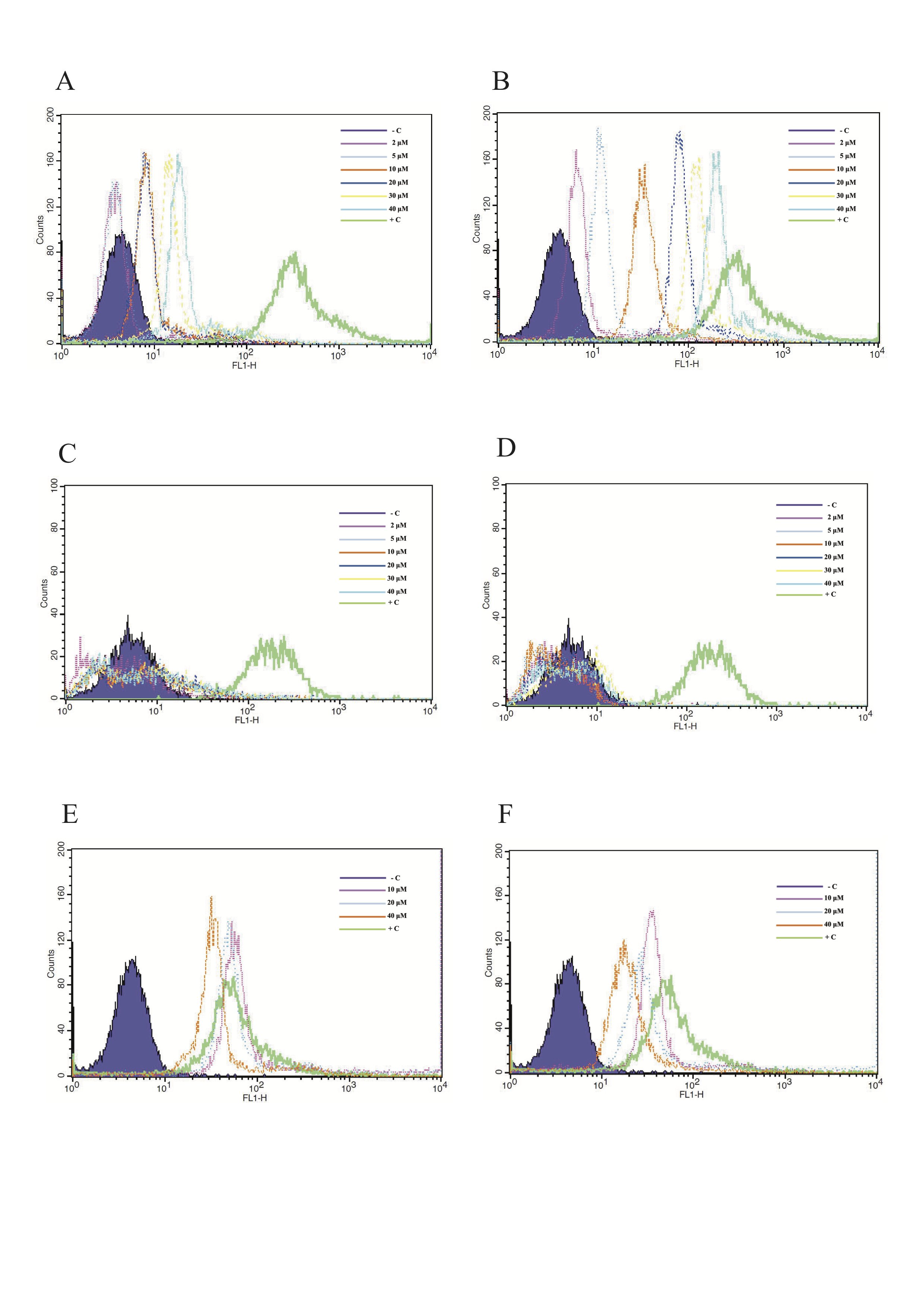

Supplement: S2 Fig — For various peptide concentrations, (A) shows the binding of fluorescent p_E2-site1 on Huh-7 cells, (B) shows the binding of fluorescent p_E2-site2 on Huh-7 cells, (C) shows the binding of fluorescent p_E2-site1 on rat PC12 cells, (D) shows the binding of fluorescent p_E2-site2 on rat PC12 cells, (E) shows the fluorescence histograms of the inhibitions of fluorescent anti-CD81 antibodies targeting Huh 7 cells by p_E2-site1 peptides, and (F) shows the fluorescence histograms of the inhibitions of fluorescent anti-CD81 antibodies targeting Huh 7 cells by p_E2-site2 peptides. In all experiments, untreated cells were used as negative controls (−C), and the cells treated with fluorescent-labelled anti-CD81 antibodies were used as positive controls (+C). (TIFF) [file pone.0177383.s002.tiff]

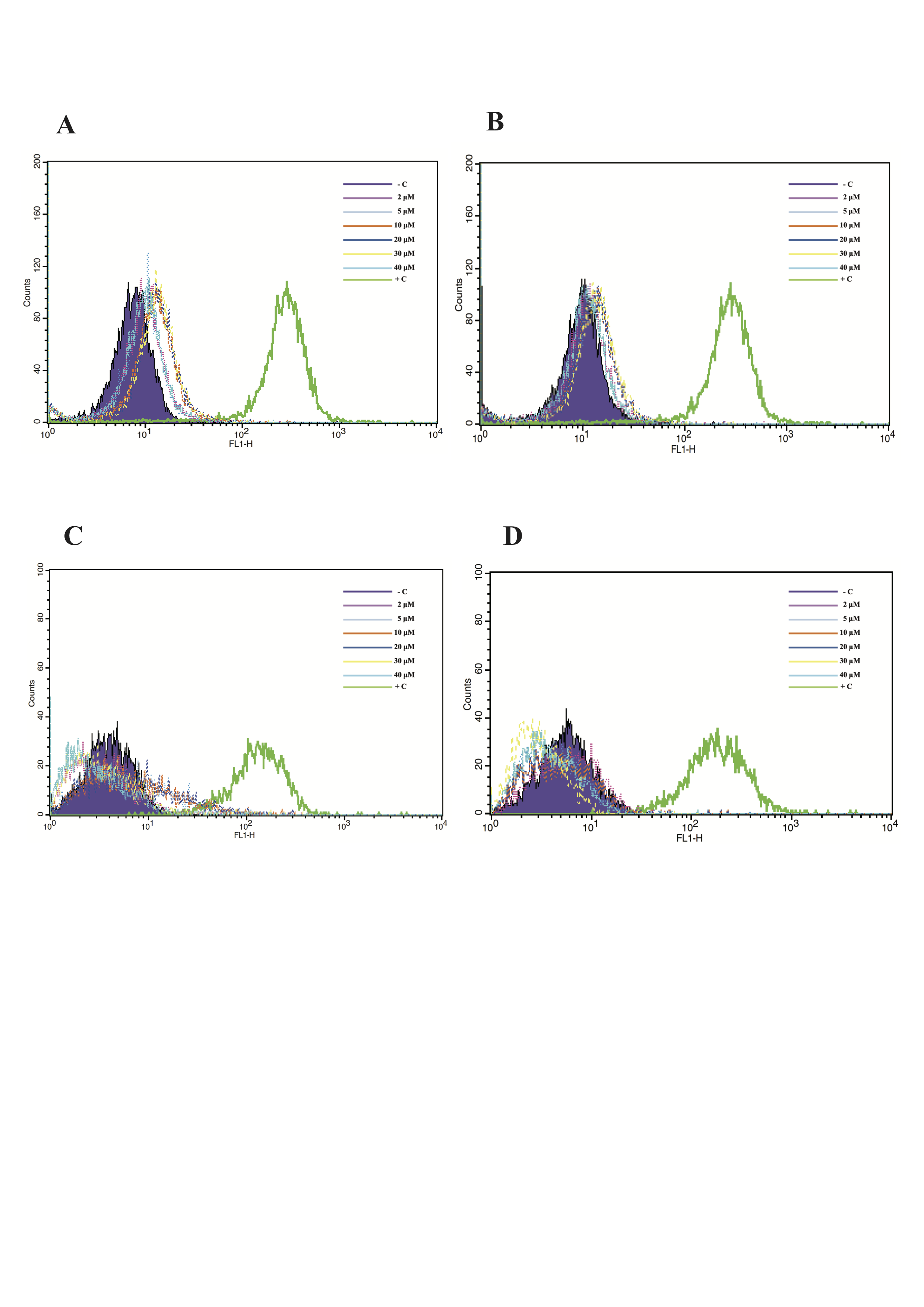

Supplement: S3 Fig — For various peptide concentrations, (A) shows the binding of fluorescent p_m_E2-site1 on Huh-7 cells, (B) shows the binding of fluorescent p_m_E2-site2 on Huh-7 cells, (C) shows the binding of fluorescent p_m_E2-site1 on rat PC12 cells, (D) shows the binding of fluorescent p_m_E2-site2 on rat PC12 cells. In all experiments, untreated cells were used as negative controls (−C), and the cells treated with fluorescent-labelled anti-CD81 antibodies were used as positive controls (+C). No dose dependent increase in fluorescence on the cells was observed when the fluorescent mutant peptides were added in different concentrations, indicating the mutant peptides were not able to bind to the CD81 presenting cells. (TIFF) [file pone.0177383.s003.tiff]
