# Supplementary material for: A Sequence in the loop domain of hepatitis C virus E2 protein identified in silico as crucial for the selective binding to human CD81
Source: PLoS One. 2017 May 8;12(5):e0177383. doi: 10.1371/journal.pone.0177383 (PMC5421814; doi:10.1371/journal.pone.0177383)
Supplement: S1 Table — The mutated amino acids in the HCV E2 mutant peptides (p_m_E2-site1 and p_m_E2-site2) are designed based on the mutation studies [11,13]. (DOCX) [file pone.0177383.s004.docx]

S1 Table.

| **Name** | **Peptide Sequence** |
| --- | --- |
| **p_random-25** | CPLNGSTVYGHLRHCLSCSGTMVKF |
| **p_random-18** | SENHLRHCLSCSKCRKEM |
| **p_m_E2-site1** | ^422^INSAGLNCNASLNTAWLGGLFAQ^444^ |
| **p_m_E2-site2** | ^521^RSAGATASAAANDTAVF^537^ |
